# Data on phosphorous concentration of rivers feeding into Taham dam in Zanjan, Iran

**DOI:** 10.1016/j.dib.2018.01.068

**Published:** 2018-01-31

**Authors:** Mohamadreza Masoudinejad, Mansour Ghaderpoori, Ahmad Zarei, Jamal Nasehifar, Alireza Malekzadeh, Jalil Nasiri, Afshin Ghaderpoury

**Affiliations:** a Safety Promotion and Injury Prevention Research Center (SPIPRC), Shahid Beheshti University of Medical Sciences, Department of Environmental Health Engineering, Tehran, Iran; b Nutritional Health Research Center, Lorestan University of Medical Sciences, Khorramabad, Iran; c Department of Environmental Health Engineering, School of Health and Nutrition, Lorestan University of Medical Sciences, Khorramabad, Iran; d Department of Environmental Health Engineering, School of Public Health, Gonabad University of Medical Sciences, Gonabad, Iran; e Department of Environmental Health Engineering, School of Public Health, Zanjan University of Medical Sciences, Zanjan, Iran; f Shahid Beheshti University of Medical Sciences, Tehran, Iran

**Keywords:** Eutrophication, Phosphorous, Rivers, Sewage, Seasons

## Abstract

Due to the great possibility of water contamination of many rivers by human activities in Iran, the study of water quality is crucial for water resource protection and human health. High level of phosphorous is the main reason for eutrophication of freshwater systems. The main aim of this study was to investigate the concentration of phosphorus in the rivers feeding into Taham dam in Zanjan, using GIS software. 40 sampling stations were selected along Taham and Ghalharod Rivers with respect to sewage discharge points and feeding characteristics of water entering to Taham dam. In total, 160 water samples were taken from rivers with regard to precipitation season in two different periods from winter 2014 to spring 2015. The obtained data were analyzed using SPSS and ArcView GIS. The findings showed that 15% of the studied stations had phosphorous levels higher than acceptable levels set by EPA. The highest levels of phosphorous contamination were observed in stations No. 145, 154, 155, 161, 166 and 168. The elevated concentrations of phosphorous in the rivers can be responsible for the eutrophication of Taham dam reservoir.

**Specifications Table**

**Table:** 

Subject area	*Chemistry, biology*
More specific subject area	*Water monitoring and quality*
Type of data	*Figure*
How data was acquired	*The levels of phosphorus in water samples were determined using a DR-5000 UV–vis spectrophotometer at wavelength 470nm (HACH, Germany).*
Data format	*Raw, analyzed,*
Experimental factors	*After selection of sampling stations, the coordinates of stations were recorded using a GPS system (Garmin GPSMAP 76CSX) and subsequently a map of the GPS points were created. The final transfer of data was done by Arc GIS.*
Experimental features	*Measuring the concentration of phosphorus in the feeding rivers of Taham dam which provides the water demands of Zanjan city*
Data source location	*Zanjan city, Zanjan province, west of Tehran, Iran*
Data accessibility	*Data are included in this study and supplemented excel file*

**Value of the data**

• Contamination of water in rivers and streams can be considered as an index of environment pollution by human activities.
• Because rivers are the only water resources which pass long distances among cities, towns, industrial and agricultural lands and therefore, there is a great possibility of their contamination by various contaminants from anthropogenic activities.
• High level of phosphorous is the main reason for eutrophication of freshwater systems (9). Eutrophication can be occurred due to the existence of nitrogen and phosphorous compounds in water which changes the water ecosystem and causes many problems in water bodies.
• High levels of phosphorous in water are not poisonous directly, and there are no reports in the literature regarding direct poisonous or harmful effects associated with elevated concentrations of phosphorus in the human body but in general decrease the concentrations of calcium and magnesium in human blood.

## 1 Data

**Fig. 1:**
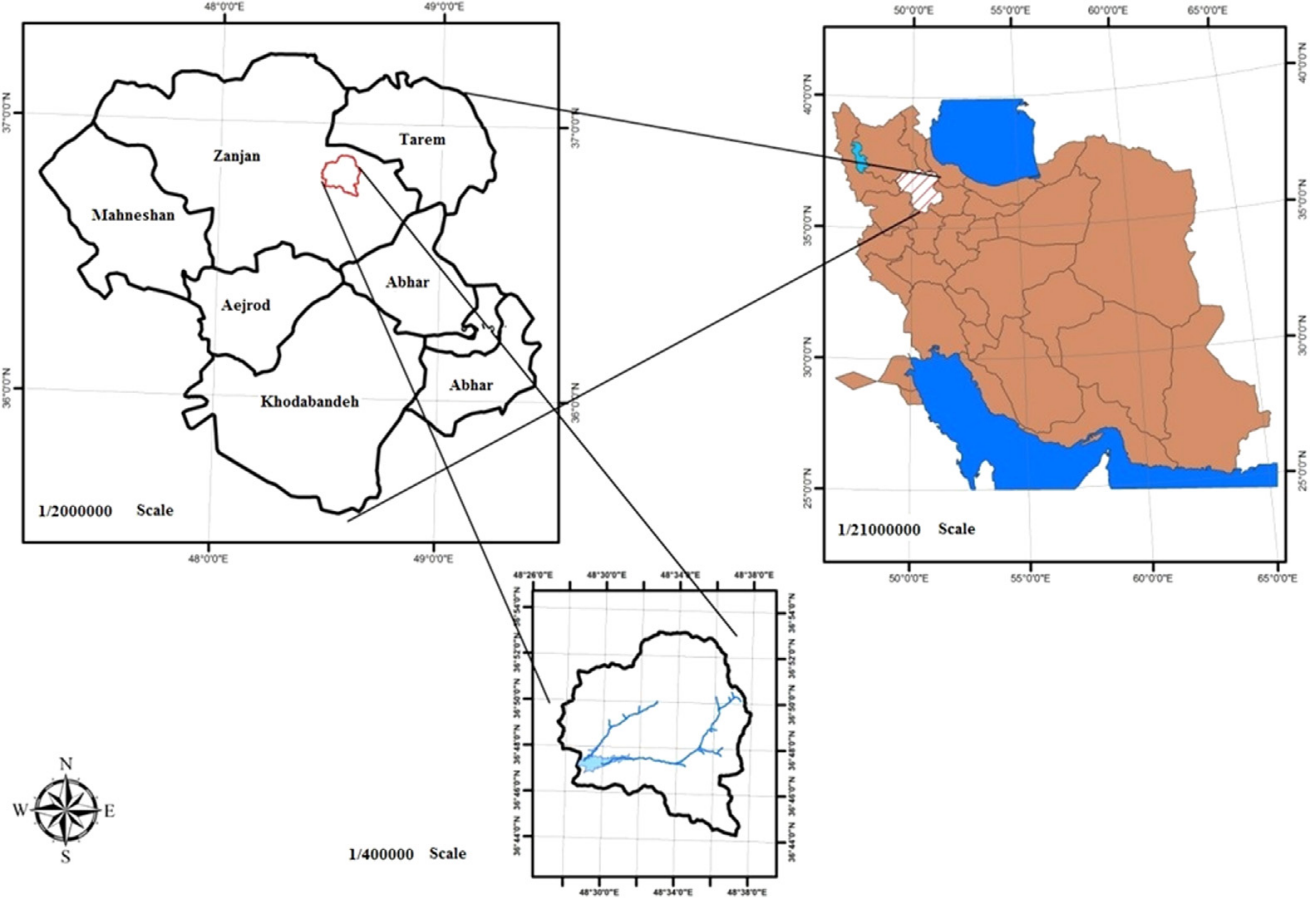
Study area location.

Zanjan city is located in 330 km from west of Tehran with a total population of 411,001 inhabitants. The study area lies in Longitudes 48°29′E and Latitudes 36°40′N with an elevation of 1650 m above sea level. The location of the Zanjan city shows in Fig. 1. Taham dam is located in 15 km in the north–west of Zanjan city and 8 km downward of Taham town. This dam provides distribution water of Zanjan city.

## 2 Experimental design, materials, and methods

**Fig. 2:**
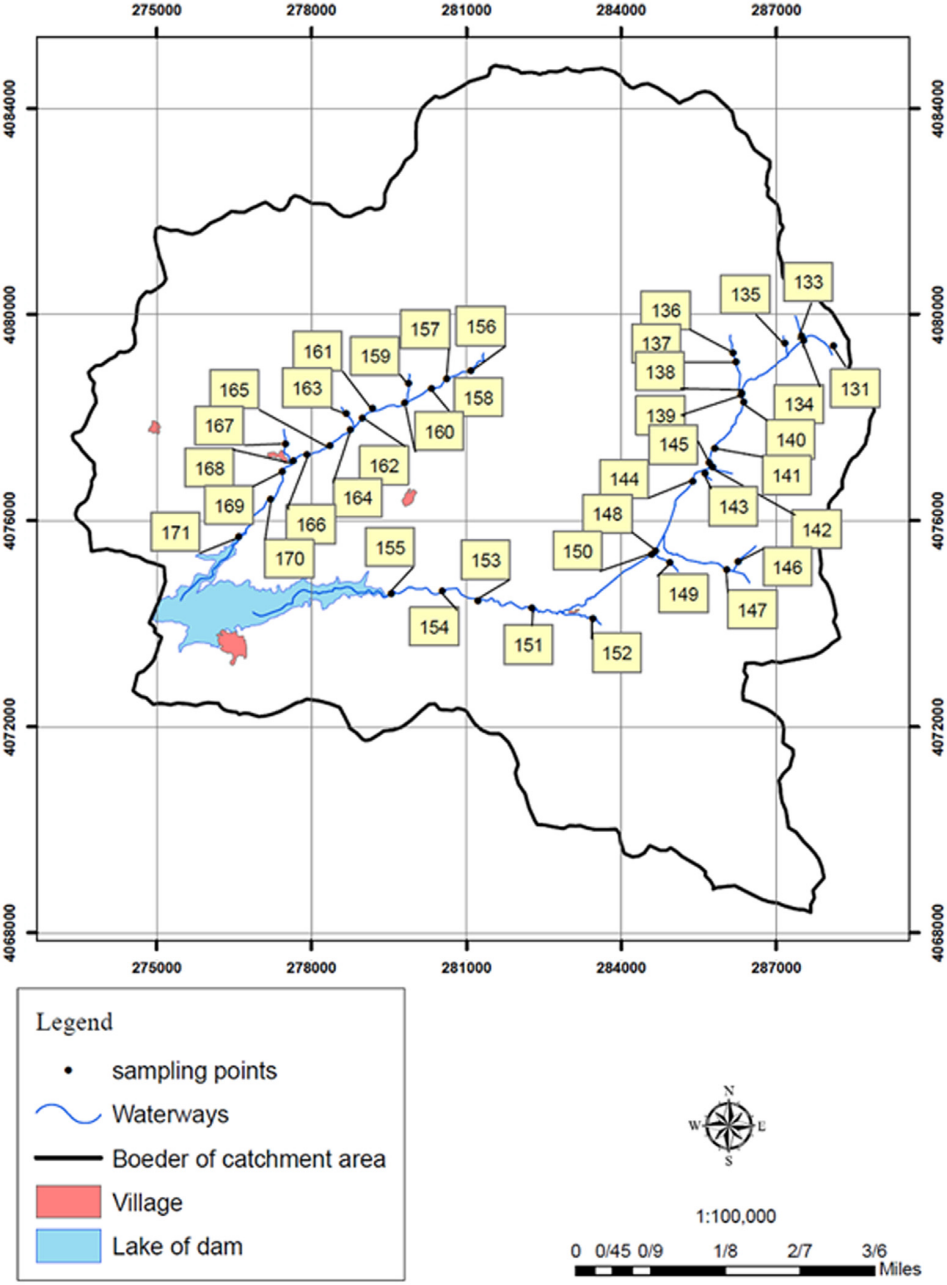
Study area and sampling stations.

**Fig. 3:**
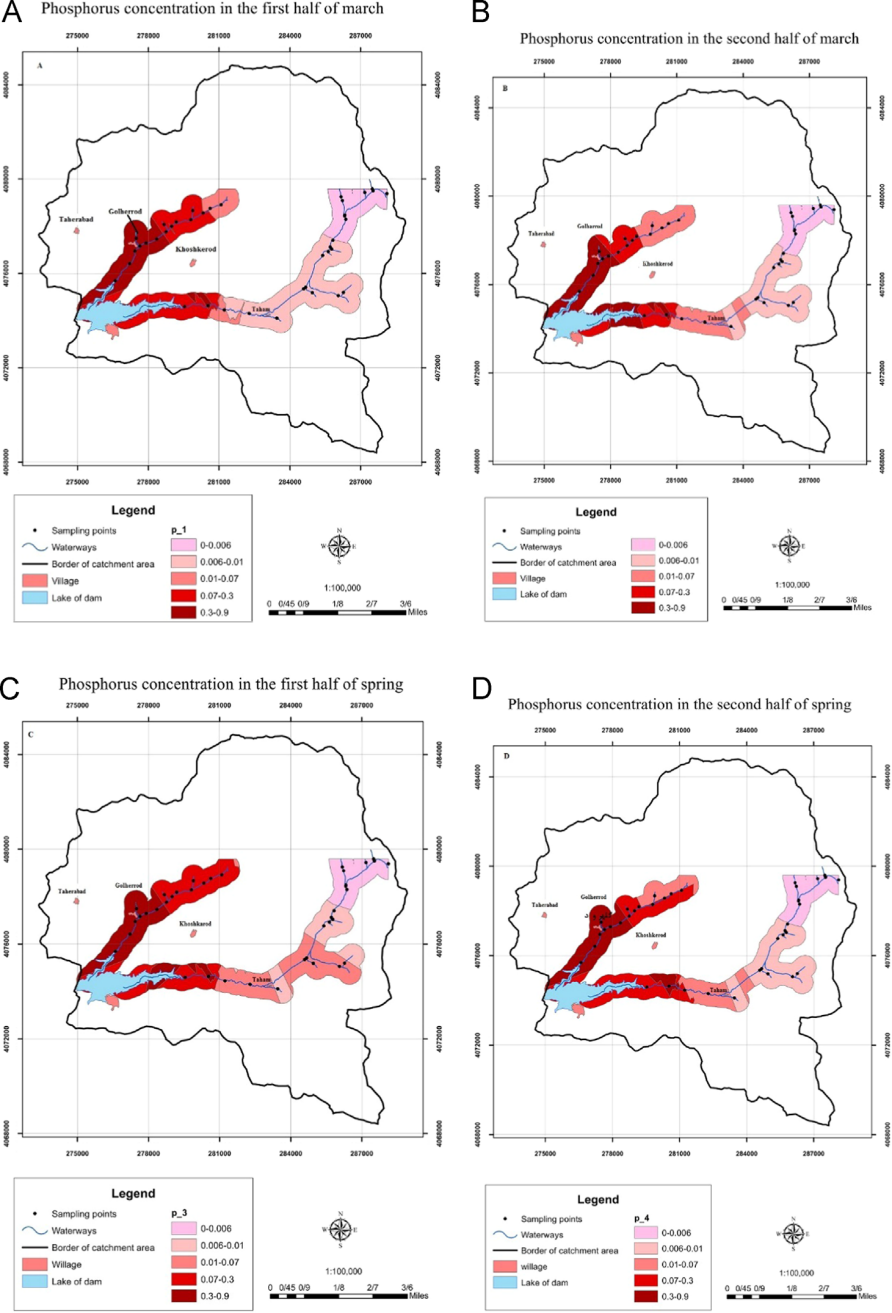
Distribution of phosphorous levels in a. February 2014, b. March 2014, c. first half of spring season 2015 and, d. second half of spring season 2015 in the study area.

This work was a cross-sectional study. In general, 40 sampling stations were selected along Taham and Ghalharod rivers with respect to the location of contaminant sources, the point of sewage discharge and the ease of sampling. A number of 160 samples were collected from two rivers in the period of farming and wet season when runoff from agricultural fields washing away inorganic fertilizers into the rivers from two periods in winter 2014 and spring. Fig. 2. shows the study area. The maximum acceptable level of phosphorous in drinking water is set 0.2, 0.54 and 0.2 mg/l by Canadian national health, European Economic Community, and EPA, respectively. The levels of phosphorous in the sampling stations of the rivers are shown in Fig. 3. As shown in Fig. 3a–d, the levels of phosphorous changed along the rivers. Accordingly, the highest levels of phosphorous in Taham River were observed in sampling stations No. 155, 154 and 145 with 0.39, 0.21 and 0.36 mg/l, respectively. The investigation of phosphorous levels in Taham and Ghalharod Rivers using GIS system showed that 15% of the studied stations had levels higher than acceptable levels set by EPA [1], [2], [3], [4], [5], [6], [7], [8], [9], [10], [11], [12], [13], [14], [15], [16], [17], [18], [19].
