# Supplementary material for: Data on phosphorous concentration of rivers feeding into Taham dam in Zanjan, Iran
Source: Data Brief. 2018 Jan 31;17:564–9. doi: 10.1016/j.dib.2018.01.068 (PMC5852270; doi:10.1016/j.dib.2018.01.068)
Supplement: Supplementary file 1 — Supplementary material [file mmc1.docx]

Dear Professor,

Editor of **Data in Brief**

The authors of this article declare that they have no conflict of interests.

**Application of GIS in the investigation of phosphorous pollution of rivers feeding into Taham dam in Zanjan, Iran**

**Mohammadreza Masoudinejad, Mansour Ghaderpoori, Ahmad Zarei, Jamal Nasehifar, Alireza Malekzadeh, Jalil Nasiri, Afshin Ghaderpoury**
